# Seed-Specific Expression of the Arabidopsis *AtMAP18* Gene Increases both Lysine and Total Protein Content in Maize

**DOI:** 10.1371/journal.pone.0142952

**Published:** 2015-11-18

**Authors:** Yujie Chang, Erli Shen, Liuying Wen, Jingjuan Yu, Dengyun Zhu, Qian Zhao

**Affiliations:** State Key Laboratory of Agricultural Biotechnology, College of Biological Sciences, China Agricultural University, Beijing, China; National Taiwan University, TAIWAN

## Abstract

Lysine is the most limiting essential amino acid for animal nutrition in maize grains. Expression of naturally lysine-rich protein genes can increase the lysine and protein contents in maize seeds. *AtMAP18* from *Arabidopsis thaliana* encoding a microtubule-associated protein with high-lysine content was introduced into the maize genome with the seed-specific promoter *F128*. The protein and lysine contents of different transgenic offspring were increased prominently in the six continuous generations investigated. Expression of *AtMAP18* increased both zein and non-zein protein in the transgenic endosperm. Compared with the wild type, more protein bodies were observed in the endosperm of transgenic maize. These results implied that, as a cytoskeleton binding protein, AtMAP18 facilitated the formation of protein bodies, which led to accumulation of both zein and non-zein proteins in the transgenic maize grains. Furthermore, F_1_ hybrid lines with high lysine, high protein and excellent agronomic traits were obtained by hybridizing T_6_ transgenic offspring with other wild type inbred lines. This article provides evidence supporting the use of cytoskeleton-associated proteins to improve the nutritional value of maize.

## Introduction

Maize (*Zea mays* L.) is one of the most important cereal foods for both humans and animals, but its nutritional value is limited because it lacks some essential amino acids in the kernel, specifically lysine [1, 2]. Efforts to improve the essential amino acid contents in maize were initiated in the mid-20th century and significant accomplishments have been made.

Many high-lysine maize varieties have been identified by genetic approaches. The most well-known mutant *opaque2* has higher lysine content than normal maize seeds [3]. The content of the Lys-poor seed storage protein zein is reduced in *o2* because the *O2* gene encodes a bZIP transcription factor that regulates zein transcription [4]. Other mutants, *opaque-7* (*o7*), *floury-2* (*fl2*) and *shrunken-4* (*sh4*), have increased proportions of lysine because of reduced zein as well [5]. *O7* encodes an acyl-activating enzyme-like protein that affects storage protein synthesis, particularly the 19- and 22-kD α-zeins [6], while *fl2* encodes a defective 22-kD α-zein, thus improving lysine content in a different way [7].

Although the *o2* mutant has high lysine content, its inferior agronomic characteristics including reduced protein content and soft endosperm are a barrier to commercial utilization [2]. Subsequently, *opaque2*-derived quality protein maize (QPM) lines appeared to overcome this drawback. QPM has the kernel properties and yield potential of normal maize and maintains the increased lysine content of *o2* [8]. Effective *o2* modifier genes, such as the 27-kD γ-zein gene, which suppresses the soft and starchy endosperm characteristics and preserves the high-lysine content, usually provide the background for QPM [9, 10].

The development of transgenic techniques has offered new opportunities for further studies on high-lysine maize [2]. RNA interference (RNAi) is a useful technology to down-regulate dominant endogenous genes [11]. High-lysine maize lines can be created with α-zein RNAi mutants to improve the lysine content of transgenic lines by reducing α-zein mRNA and increasing the non-zein fraction [12, 13]. One study showed that the lysine content was increased more than 25% in vitreous kernels from α-zein RNAi mutants crossed with a QPM line [13].

Manipulating the regulatory steps that control lysine synthesis and metabolism has long been considered an alternative strategy for producing high-lysine plants. Aspartate kinase (AK) and dihydrodipicolinate synthase (DHDPS), two key enzymes in the lysine synthesis pathway, are both feedback inhibited by lysine [2]. There are two common methods to increase lysine content: one is expression of the lysine-insensitive *AK* or *DHDPS* genes in transgenic plants, the other is inducing mutations using mutagens [14–16]. Lysine accumulation can also be enhanced by reducing the activity of Lys-ketoglutarate reductase/saccharopine dehydrogenase (LKR/SDH), a bifunctional enzyme in the lysine catabolism pathway. Generally, the degradation of lysine is blocked by knockout mutation or seed-specific RNAi-mediated suppression of the *LKR/SDH* gene [11, 15, 17–18].

Cultivating valuable maize germplasm with the high protein, high lysine and hard endosperm traits is still a breeding challenge [1]. Recent progress in enhancing the nutritional value of maize was made through the introduction of heterologous genes encoding proteins rich in essential amino acids. Expressing Sb401, a pollen-specific protein with high lysine content from *Solanum berthaultii*, in maize kernels resulted in prominent increases in the total protein and lysine contents [19]. *SBgLR*, a homolog of *Sb401*, was introduced into maize, and the protein and lysine contents of *SBgLR* transgenic plants showed more than 30% increases compared with untransformed plants [20]. When *SBgLR* and *TSRF1* (an ethylene responsive transcription factor gene from tomato) were co-transformed into maize, the protein and lysine contents of transgenic maize seeds also increased significantly [21]. In addition, Yue et al. [22] cloned a lysine-rich protein gene, *GhLRP*, from cotton and seed-specifically expressed it in maize, clearly increasing the lysine content of transgenic maize seeds relative to the wild-type.

*MICROTUBULE-ASSOCIATED PROTEIN 18* (*AtMAP18*) is located on chromosome 5 of the *Arabidopsis* genome (*At5g44610*). It encodes a polypeptide of 168 amino acid residues that contains seven repeated motifs of V-E-E-K-K. The encoded protein has high lysine content (20.3%, w/w). It has been demonstrated that AtMAP18 has a destabilizing effect on cortical microtubules (MTs) and influences actin organization. AtMAP18 plays an important role in regulating directional cell growth by modulating actin filaments [23, 24].

To investigate whether *AtMAP18* could increase the lysine content in maize kernels, it was introduced into the maize genome with a seed-specific promoter, *F128* [25]. Accumulation of AtMAP18 resulted in simultaneous increases of lysine and protein contents in transgenic maize seeds. Through further study, we found that the expression of *AtMAP18* promoted protein body (PB) formation and increased the accumulation of both zein and non-zein proteins in maize grains, which might be a possible reason for the enhancement of the nutritional value of transgenic maize.

## Materials and Methods

### Plasmid construction and maize transformation

**Fig 1:**
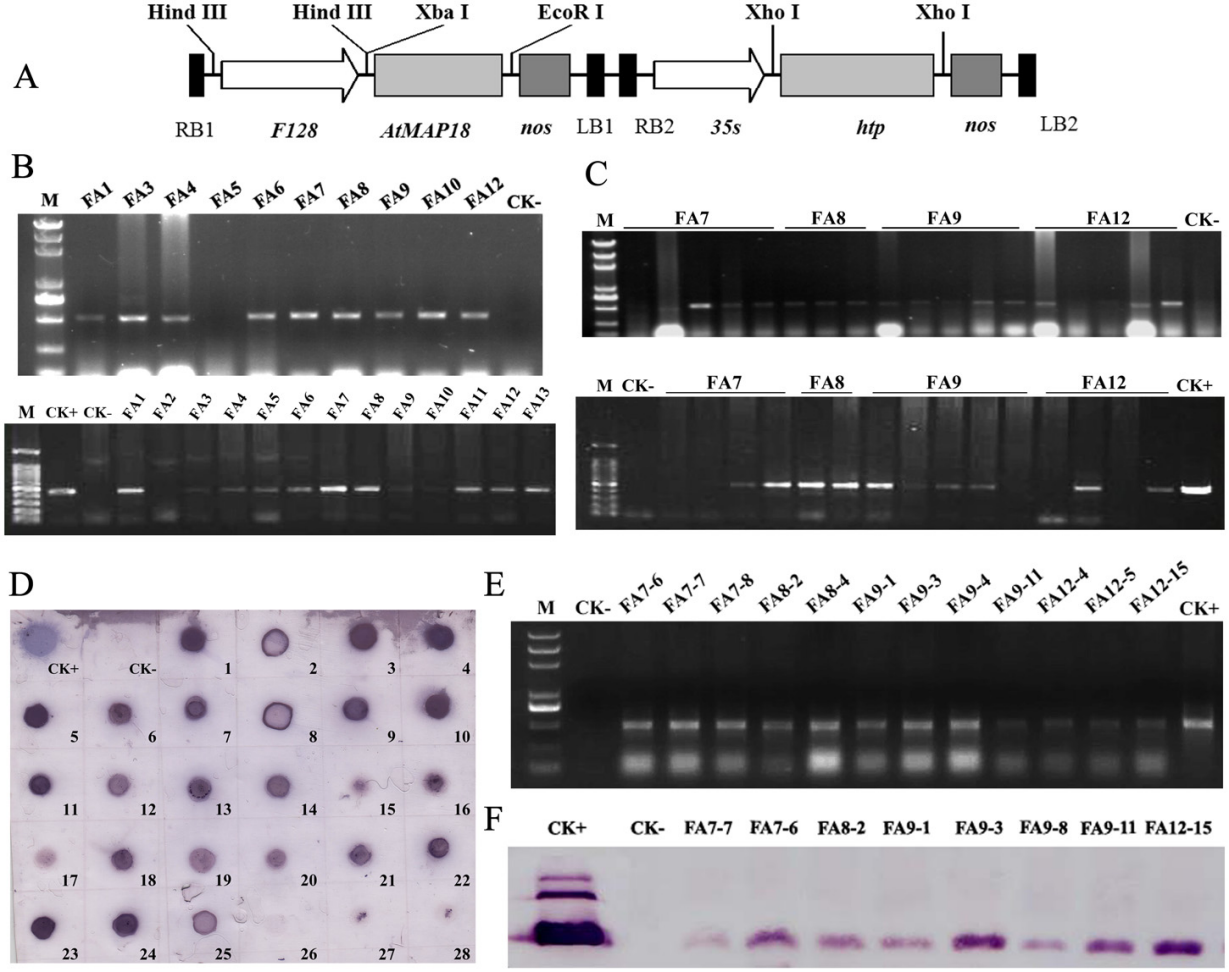
Molecular analysis of transgenic events. (A) Binary vector pSB130-*AtMAP18*. (B) PCR analysis of T_0_ transgenic lines. PCR was carried out using primers corresponding to the *AtMAP18* sequence (top) and *hpt* sequence (bottom). M: DNA markers, CK−: wild-type (hybrid 08×178), CK+: plasmid pSB130-*AtMAP18*. (C) PCR analysis of T_1_ transgenic lines. PCR was carried out using primers corresponding to the *AtMAP18* sequence (top) and *hpt* sequence (bottom). (D) Dot blot analysis of transgenic lines. Blot 1–5: FA7; blot 6–11: FA8; blot 12–18: FA9; blot 19–28: FA12. FA1–FA13: transgenic lines. (E) RT-PCR analysis of the *AtMAP18* transcript in the transgenic lines. (F) Western blot analysis. CK+: AtMAP18 protein in *Escherichia coli*.

*AtMAP18* was isolated from *Arabidopsis thaliana* by RT-PCR, and then sub-cloned into the *Pst*I/*Eco*RI site of the double T-DNA binary vector pSB130 (kindly provided by Prof. Sai-Ming Samuel Sun, The Chinese University of Hong Kong) to yield the expression vector pSB130-*AtMAP18*. In this construct, the *AtMAP18* coding sequence was transcriptionally fused to the *F128* promoter and *nos* region of T-DNA1, and the selectable marker gene *hygromycin phosphotransferase* (*hpt*) driven by the *CaMV35S* promoter was contained in T-DNA2 (Fig 1A). For plant transformation, this plasmid was transferred into *A*. *tumefaciens* strain LBA4404. The inbred maize lines 08 and 178 were cross-pollinated to produce the hybrid line 08×178, and immature embryos (1.5–2.0 mm) of this hybrid line at 10–12 DAP (days after pollination) were used for *Agrobacterium*-mediated transformation as previously described [26].

### DNA extraction and PCR analysis

Transgene insertion in regenerated plants was confirmed by PCR amplification. Genomic DNA was isolated from fresh leaves using a cetyltrimethyl ammonium bromide (CTAB) method [27]. PCR was carried out using 50 ng DNA, 10 μL of 2× PCR mix buffer (GenStar, Beijing, China) and 0.5 μL of each primer (10 μM/L) in a 20 μL volume. The forward (5′-ATGGGTTATTGGAAGTCGAAGG-3′) and reverse (5′-TCAAGCCTTTTGTGGCGCAGCC-3′) primers corresponding to the *AtMAP18* sequence amplified a fragment of 500 bp. The forward (5′-TCGGCTCCAACAATGTCCTG-3′) and reverse (5′-CGGTCGGCATCTACTCTATTCC-3′) primers corresponding to the *hpt* sequence amplified a fragment of 480 bp. The amplification included 30 cycles of 94°C for 30 s, 54°C for 30 s and 72°C for 30 s. The PCR products were analyzed on 0.8% agarose gels.

### Dot blot

A total of 10 μg genomic DNA was denatured by heating and blotted onto a Hybond TM-XL membrane (Amersham, Bath, UK) soaked with 2× SSC. The membrane was then dried and baked at 80°C for 2 h. Hybridization was performed according to the manufacturer’s protocol for the Dig High Primer DNA Labeling and Detection Starter Kit I (Roche, Basel, Switzerland)

### RT-PCR and real-time PCR

Total mRNA was isolated from T_1_ immature endosperm at 20 DAP using Trizol Reagent (CWBIO, Beijing, China). The RNA was treated with RNase-free DNase I (TaKaRa, Otsu, Japan) and purified. For RT-PCR, 3 μg of total mRNA was used for cDNA synthesis according to the manufacturer's protocol (Promega A3500, Madison, WI, USA). Amplifications were performed in a total volume of 20 μL containing 100 ng cDNA, 2× PCR mix buffer (GenStar, Beijing, China) and 0.5 μM specific primers (forward 5′-ATGGGTTATTGGAAGTCGAAGG-3′; reverse (5′-TCAAGCCTTTTGTGGCGCAGCC-3′) corresponding to the *AtMAP18* sequence; a fragment of 500 bp was amplified.

qRT-PCR was performed using 100 ng cDNA, 10 μL of 2× UltraSYBR Mixture (CWBIO, Beijing, China) and 0.5 μM of each primer in a 20 μL volume. Specific forward (5’- AGGAGGTCGTCGTGAAAAC-3’) and reverse (5’- GCTTCTTCTCCTCCGCAGC-3’) primers were used to amplify a 162 bp internal fragment of *AtMAP18*. Additionally, forward (5’-GAGCATGGCATTCAGGCTGACG-3’) and reverse (5’-TCAACAAAAACAGCACGGGGCA-3’) primers were designed to amplify a region of the actin transcript as an internal control.

### Western blot

Dried kernels were ground and about 0.1 g of powder was defatted with 1 mL hexane for 1 h. Then, 1 mL extraction buffer (20 mM Tris-HCl, pH 8.0, 5 mM EDTA, 0.05% SDS, 10 mM DTT, 1 mM PMSF) was added and the sample was vortexed for 10 min. After centrifugation at 13,000× *g* for 10 min, 10 μL of the supernatant was separated on 12% SDS-PAGE and transferred onto a PVDF membrane (Millipore Corporation, Darmstadt, Germany). The membrane was blocked with 3% BSA, and incubated with anti-*AtMAP18* antibody (1:5000) and then a goat anti-rabbit alkaline phosphatase-conjugated IgG antibody as a secondary antibody (1:5000 Promega, Madison, WI, USA). Protein bands were visualized using a NBT/BCIP reaction kit (Promega S380C; S381C, Madison, WI, USA).

### Preparation of the anti-*AtMAP18* antibody

The anti-*AtMAP18* antibody was obtained by the following program. The recombinant AtMAP18 protein was purified as previously described [28] and injected into a rabbit to elicit antiserum. The antiserum was purified using the Protein A resin column and the cyanogen bromide resin column (Amersham, WI, USA). The anti-*AtMAP18* antibody was used as a primary antibody in Western blot.

### Protein, lysine, zein and non-zein contents analysis

Total protein content analysis was based on Kjeldahl determination. About 5 g of mature kernels were ground to powder for total nitrogen detection (national standard GB2905-82, Beijing Academy of Agriculture and Forestry Science). The conversion factor between nitrogen content and protein content is 6.25. Lysine content was analyzed by the ninhydrin method as described previously [22]. All measurements were replicated at least three times.

Zein and non-zein proteins were extracted from 50 mg endosperm flour according to a previously described method [22]. Quantitative protein determination of the total extract, zein and non-zein fractions was performed with a BCA protein assay kit (Pierce, Rockford, IL, USA). Protein samples (10 μL) were used for SDS-PAGE analysis with 12% polyacrylamide gels, and the gels were stained with Coomassie brilliant blue R250 (Amresco, Solon, OH, USA).

### Transmission electron microscopy and scanning electron microscopy

For transmission electron microscopy (TEM), 20-DAP transgenic T_6_ and wild-type kernels were fixed with potassium phosphate buffer (1% glutaraldehyde, 4% paraformaldehyde and 5 mM EGTA, pH 6.8) at 4°C overnight and post-fixed in fixation buffer containing 1% osmium tetroxide at 4°C overnight. Fixed slices were dehydrated in an ethanol gradient up to 100%. The samples were embedded in Spurr and LR White resin for ultrathin sectioning. The thin sections (90 nm) were collected on formvar-coated nickel grids, stained with 2% uranyl acetate and 2.66% lead citrate, and rinsed three times in ddH_2_O. The sections were visualized using a Hitachi 7500 electron microscope (Hitachi, Tokyo, Japan) operated at 80 kV.

For scanning electron microscopy (SEM), Mature transgenic T_6_ and wild-type kernels were dissected, fixed on a brass disk, and covered with gold/palladium by an ion coater (EIKO IB.3, Japan) The central region of the starchy endosperm was observed by SEM (JEOL, Tokyo, Japan).

### Agronomic quality measurement

For agronomic quality analysis, ear length, bald tip length, ear diameter, numbers of ear rows and 100-kernel weight were measured. Each agronomic trait was measured three times in mature maize of the transgenic lines and WT (08×178). The kernel phenotypes of FA7, FA9, FA12 and the WT were observed with a stereoscopic microscope (OLYMPUS SZ61, Tokyo, Japan).

### Ethics Statement

The Committee of Experiment Animals of China Agricultural University approved the protocols we used in our study for antibody production. The rabbit was raised in standardized pathogen-free conditions in the Animal Care Facility at Beijing B&M Biotech Co., Ltd. Blood was drawn from the marginal ear vein under anesthesia to ameliorate suffering.

## Results

### Maize transformation and molecular analysis

*AtMAP18* was isolated from *A*. *thaliana* by RT-PCR. It encoded a polypeptide of 168 amino acid residues containing seven repeated motifs of V-E-E-K-K. Immature embryos of the maize hybrid 08×178 were used as recipient materials for transformation, and the double T-DNA expression vector pSB130-*AtMAP18* containing *AtMAP18* and *hpt* was used for *Agrobacterium*-mediated transformation. *AtMAP18* was driven by the seed-specific promoter *F128* (Fig 1A). A total of 108 regenerated plants were obtained after hygromycin selection and induced redifferentiation. Among these plants, 35 contained the target gene *AtMAP18* and the selectable marker gene *hpt* according to PCR detection (Fig 1B). The T_0_ transgenic plants were self-pollinated to obtain T_1_ progeny. PCR results showed that 13 lines contained the *AtMAP18* gene and lacked the *hpt* gene after gene separation in the T_1_ transgenic lines (Fig 1C). These results indicated that double T-DNA application was an effective way to obtain marker-free transgenic plants. In addition, PCR analysis of the T1 plants also showed that 20 T_0_ transgenic lines exhibited segregation at a ratio of approximately 3:1 (data not shown), implying a single copy of *AtMAP18* gene was integrated in these lines. The copy number of some homozygous transgenic lines also have been confirmed by real-time PCR assay (S1 Table). Moreover, signals were also detected in the T_1_ transgenic lines by dot blot assay (Fig 1D). To determine whether *AtMAP18* was transcribed in the transgenic lines, we amplified the coding region of the *AtMAP18* gene using RT-PCR. An amplified product of the expected size (500 bp) was detected from T_1_ transgenic endosperms, but not from the wild type (Fig 1E), which indicated the *AtMAP18* gene was successfully transcribed in maize seeds. Furthermore, AtMAP18 accumulation in maize seeds was analyzed by Western blotting. Specific protein bands were detected from T_1_ mature kernels, while no bands were present in WT kernels (Fig 1F). These results demonstrated that *AtMAP18* was integrated into the maize genome and the protein could be expressed in maize seeds.

### Improved lysine and total protein contents of transgenic kernels

To explore whether AtMAP18 could improve the nutritional value of maize, the total protein and lysine contents were analyzed in different transgenic offspring. In the kernels of 31 T_1_ plants, the protein content was improved by 1.60–33.60%, with nine plants showing more than 20% improvement, while the lysine content was improved by 3.30–32.30%, with nine plants showing more than 20% improvement (Table 1). Three lines (FA7, FA9, FA12) in which the protein and lysine contents were both increased more than 20% were selected to generate subsequent generations. We continued until the T_6_ generation; both the protein and lysine contents were increased significantly relative to the WT in the following generations (Tables 2 and 3). The protein contents of FA7, FA9 and FA12 were increased by 17.83%, 16.97% and 22.72%, respectively, in T_2_, by 20.92%, 22.24% and 17.96%, respectively, in T_3_, by 26.45%, 14.05% and 17.77%, respectively, in T_4_, by 26.18%, 28.73% and 26.37%, respectively, in T_5_, and by 26.40%, 27.97% and 27.18%, respectively, in T_6_. The lysine contents of FA7, FA9 and FA12 were increased by 15.63%, 18.75% and 9.38%, respectively, in T_2_, by 16.13%, 19.35% and 22.58%, respectively, in T_3_, by 20.00%, 16.67% and 23.33%, respectively, in T_4_, by 10.00%, 33.33% and 23.33%, respectively, in T_5_, and by 18.75%, 21.88% and 25.00%, respectively, in T_6_. These results indicated that AtMAP18 improved the protein and lysine contents in transgenic maize plants and that the high-lysine and high-protein characters were heritable.

**Table 1:** Lysine and total protein contents of T_1_ kernels.

Line	Protein content (g/100 g seed)	Protein increase rate (%)	Lysine content (g/100 g seed)	Lysine increase rate (%)
FA7-6	11.88±0.21*	14.90	0.32±0.01	3.20
FA7-7	11.58±0.13*	12.00	0.30±0.02	-3.20
FA7-8	11.06±0.12	7.00	0.37±0.02*	19.40
FA7-10	11.62±0.23*	12.40	0.37±0.03*	19.40
FA7-12	11.51±0.51	11.30	0.32±0.01	3.20
FA8-1	10.51±0.35	1.60	0.35±0.03	12.90
FA8-2	10.10±0.26	-2.30	0.30±0.01	-3.20
FA8-4	12.18±0.42*	17.80	0.32±0.02	3.20
FA8-5	11.20±0.32	8.30	0.33±0.03	6.50
FA8-7	12.04±0.67	16.40	0.36±0.01*	16.10
FA9-1	13.81±0.31**	33.60	0.39±0.02**	25.80
FA9-2	13.33±0.71**	28.90	0.35±0.01	12.90
FA9-3	13.70±0.63**	32.50	0.40±0.01**	29.00
FA9-4	12.99±0.15**	25.60	0.36±0.02*	16.10
FA9-5	12.91±0.20**	24.90	0.37±0.03*	19.40
FA9-6	11.57±0.34	11.90	0.39±0.02**	25.80
FA9-7	13.05±0.30**	26.20	0.38±0.01*	22.60
FA9-8	12.59±0.26**	21.80	0.40±0.03**	29.00
FA9-9	12.52±0.47**	21.10	0.38±0.03*	22.60
FA9-10	12.82±0.70**	24.00	0.39±0.02**	25.80
FA9-12	10.08±0.41	-2.50	0.36±0.03*	16.10
FA9-13	11.18±0.22	8.10	0.41±0.01**	32.30
FA12-3	12.10±0.54*	17.00	0.34±0.02	9.70
FA12-4	12.05±0.15*	16.50	0.33±0.01	6.50
FA12-5	10.56±0.31	2.10	0.37±0.01*	19.40
FA12-6	10.72±0.27	3.70	0.37±0.02*	19.40
FA12-7	11.76±0.60*	13.70	0.39±0.02**	25.80
FA12-8	10.72±0.44	3.70	0.33±0.01	6.50
FA12-9	11.42±0.19	10.40	0.32±0.03	3.20
FA12-13	11.55±0.29	11.70	0.36±0.01*	16.10
FA12-14	10.74±0.53	3.90	0.37±0.02*	19.40
WT ^a^	10.34±0.18	/	0.31±0.01	/

Values are means ± SD from three experiments on the same line.

^a^ WT: Hybrid 08×178 in the T_1_ generation as a control.

* Significant difference between the transgenic lines and WT by Student’s *t*-test (* *p* < 0.05; ** *p* < 0.01).

**Table 2:** Protein content of T_2_, T_3_, T_4_, T_5_ and T_6_ kernels in three transgenic lines.

Line	T_2_		T_3_		T_4_		T_5_		T_6_	
	Protein content (g/100 g seed)	Protein increase rate (%)	Protein content (g/100 g seed)	Protein increase rate (%)	Protein content (g/100 g seed)	Protein increase rate (%)	Protein content (g/100 g seed)	Protein increase rate (%)	Protein content (g/100 g seed)	Protein increase rate (%)
FA7	12.29±0.70*	17.83%	11.85±0.52***	20.92%	12.24±0.29***	26.45%	12.87±0.33**	26.18%	12.88±0.47**	26.40%
FA9	12.20±0.73**	16.97%	11.98±0.67**	22.24%	11.04±0.57*	14.05%	13.13±0.54**	28.73%	13.04±0.59**	27.97%
FA12	12.8±0.75*	22.72%	11.56±0.33**	17.96%	11.40±0.40**	17.77%	12.89±0.37**	26.37%	12.96±0.39**	27.18%
WT ^a^	10.43±0.21	/	9.8±0.35	/	9.68±0.33	/	10.20±0.16	/	10.19±0.22	/

Values are means ± SD from the same lines in different generations.

^a^ WT: Hybrid 08×178 in different generations as a control.

* Significant difference between the transgenic lines and WT by Student’s *t*-test (* *p* < 0.05; ** *p* < 0.01; *** *p* < 0.001).

**Table 3:** Lysine content of T_2_, T_3_, T_4_, T_5_ and T_6_ kernels in three transgenic lines.

Line	T_2_		T_3_		T_4_		T_5_		T_6_	
	Lysine content (g/100 g seed)	Lysine increase rate (%)	Lysine content (g/100 g seed)	Lysine increase rate (%)	Lysine content (g/100 g seed)	Lysine increase rate (%)	Lysine content (g/100 g seed)	Lysine increase rate (%)	Lysine content (g/100 g seed)	Lysine increase rate (%)
FA7	0.37±0.02*	15.63%	0.36±0.02*	16.13%	0.36±0.03*	20.00%	0.33±0.02**	10.00%	0.38±0.02*	18.75%
FA9	0.38±0.03**	18.75%	0.37±0.02*	19.35%	0.35±0.01**	16.67%	0.40±0.02*	33.33%	0.39±0.03*	21.88%
FA12	0.35±0.02*	9.38%	0.38±0.01**	22.58%	0.37±0.03*	23.33%	0.37±0.01*	23.33%	0.40±0.01**	25.00%
WT ^a^	0.32±0.01	/	0.31±0.01	/	0.30±0.02	/	0.30±0.01	/	0.32±0.01	/

Values are means ± SD from the same lines in different generations.

^a^ WT: Hybrid 08×178 in different generations as a control.

* Significant difference between the transgenic lines and WT by Student’s *t*-test (* *p* < 0.05; ** *p* < 0.01).

**Fig 2:**
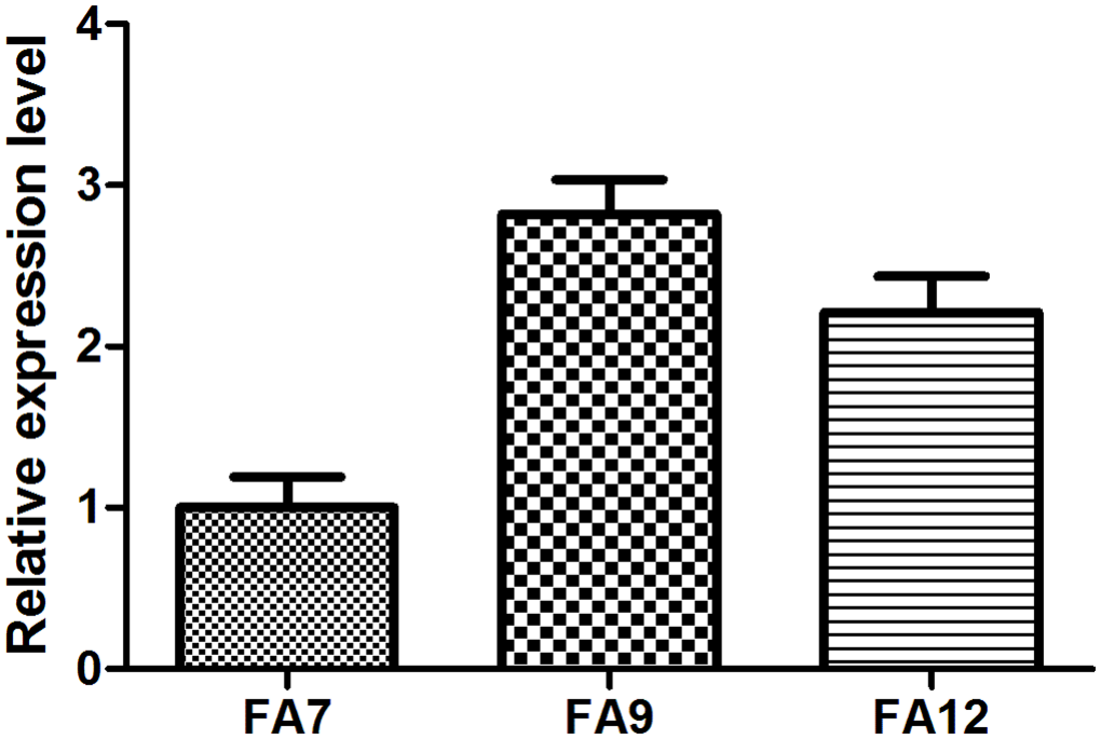
Relative expression levels of *AtMAP18* in T_6_ kernels of three transgenic lines. The transcriptional levels of *AtMAP18* in F7, F9, and F12 transgenic lines (20 days after pollination) in T_6_ were quantified by real-time PCR.

In addition, the transcriptional levels of AtMAP18 in three transgenic lines were quantified by quantitative real-time PCR (qRT-PCR). The result indicate that the transcriptional level of AtMAP18 in the F9 and F12 lines were higher than that in the F7 line, while the level was the highest in the F9 line (Fig 2). However, the lysine increase rate was the highest in the F12 line, this contradiction implied that the increase of lysine content did not only depend on expression level of *AtMAP18* gene in transgenic maize, some other factors such as genetic background may also have effects.

### Increased accumulation of both zein and non-zein protein in transgenic maize

**Fig 3:**
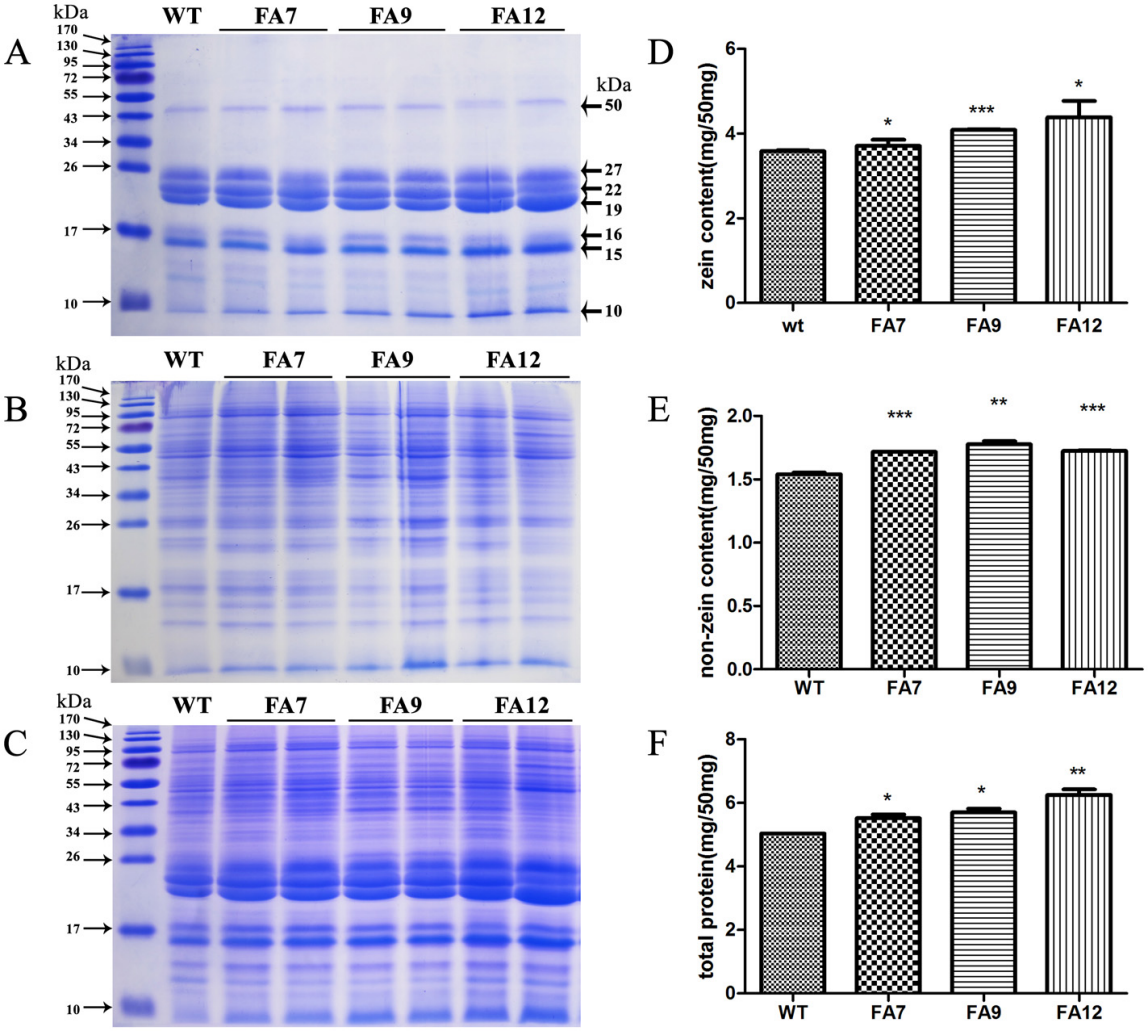
SDS-PAGE and protein content quantification assay of zein, non-zein and total protein extracted from mature kernels. (A–C) SDS-PAGE assay of zein (A), non-zein (B) and total protein (C) contents in WT, FA7, FA9 and FA12 mature kernels. (D–E) Quantification of zein (D), non-zein (E) and total protein (F) contents in WT, FA7, FA9 and FA12 mature kernels. Significant differences between the transgenic lines and WT were evaluated by Student’s *t*-test (* *p* < 0.05; ** *p* < 0.01; *** *p* < 0.001).

Ordinarily, maize seeds contain about 10% protein and approximately 70% of this comprises storage proteins. More than 60% of these storage proteins are classified as zeins; the others are non-zeins [1]. To determine whether the expression of *AtMAP18* changed the level of each protein fraction in transgenic kernels, we quantified the zein, non-zein and total proteins in T_6_ mature kernels. Compared with the WT total extract, proteins with different molecular weights were increased on the SDS-PAGE gel in T_6_ endosperm (Fig 3C). The protein contents of the total extracts from the WT, FA7, FA9 and FA12 were 5.03, 5.52, 5.70 and 6.25 mg/50 mg flour, respectively (Fig 3F). The total protein contents of the three transgenic lines were all higher than the WT.

Zeins can be divided into four subfamilies: 19- and 22-kDa α-zein, 16-, 27- and 50-kDa γ-zein, 15-kDa β-zein, and 10- and 18-kDa δ-zein [1]. Compared with the WT, the 19- and 22-kDa α-zein, 27-kDa γ-zein, 15-kDa β-zein and 10-kDa δ-zein contents were increased in the mature endosperm of FA7, FA9 and FA12 according to SDS-PAGE analysis (Fig 3A). The average contents of the zein fractions in FA7, FA9 and FA12 kernels were 3.71, 4.08 and 4.38 mg/50 mg flour, respectively, which were higher than in the WT (3.58 mg/50 mg flour) (Fig 3D).

Similar results were found in non-zein protein analysis. The non-zein protein contents of the transgenic endosperms also increased on the SDS-PAGE gel (Fig 3B). The non-zein fraction of the WT endosperm was 1.54 mg/50 mg flour, compared with 1.72, 1.78 and 1.72 mg/50 mg flour in the three transgenic lines, respectively (Fig 3E). These results suggested that seed-specific expression of *AtMAP18* increased both the zein and non-zein content in transgenic endosperm.

### Increased protein bodies in transgenic endosperms

**Fig 4:**
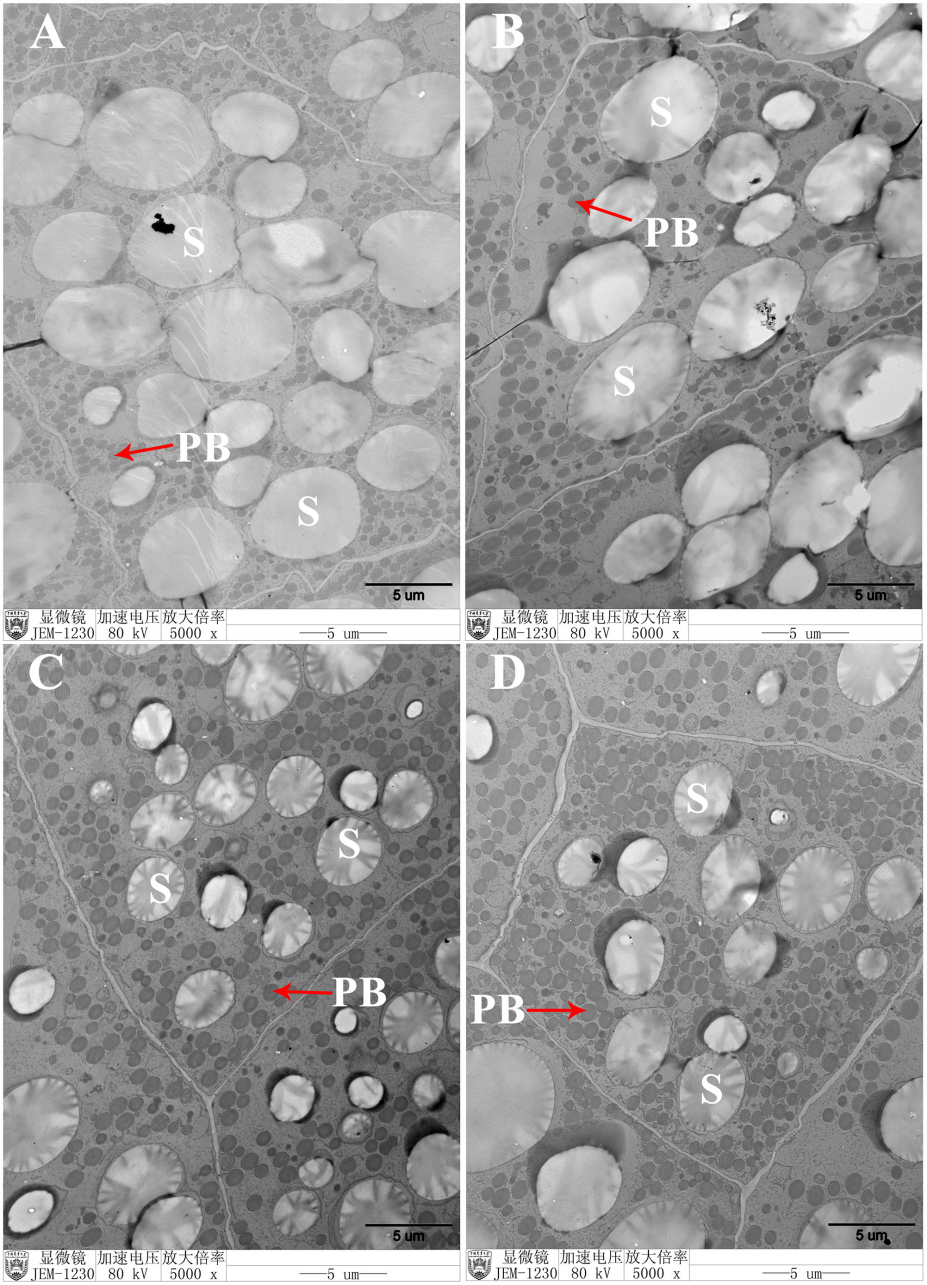
Transmission electron microscopy analysis of developing endosperm. (A–D) Endosperm cells (20 DAP) of the WT (A), FA7 (B), FA9 (C) and FA12 (D). Bar = 5 μm. PB: protein body, S: starch granule.

To investigate the mechanism by which *AtMAP18* affected the protein contents in FA7, FA9 and FA12, we compared the ultrastructure of endosperm cells at 20 DAP using TEM. In WT endosperm cells, PBs were spherical and surrounded by rough endoplasmic reticulum (RER) (Fig 4A and 4B). In T_6_ transgenic maize endosperms, the shape of PBs did not change but the number of PBs was significantly increased, and starch granules were reduced (Fig 4C and 4D). This implied that *AtMAP18* increased PB formation in developing endosperms.

**Fig 5:**
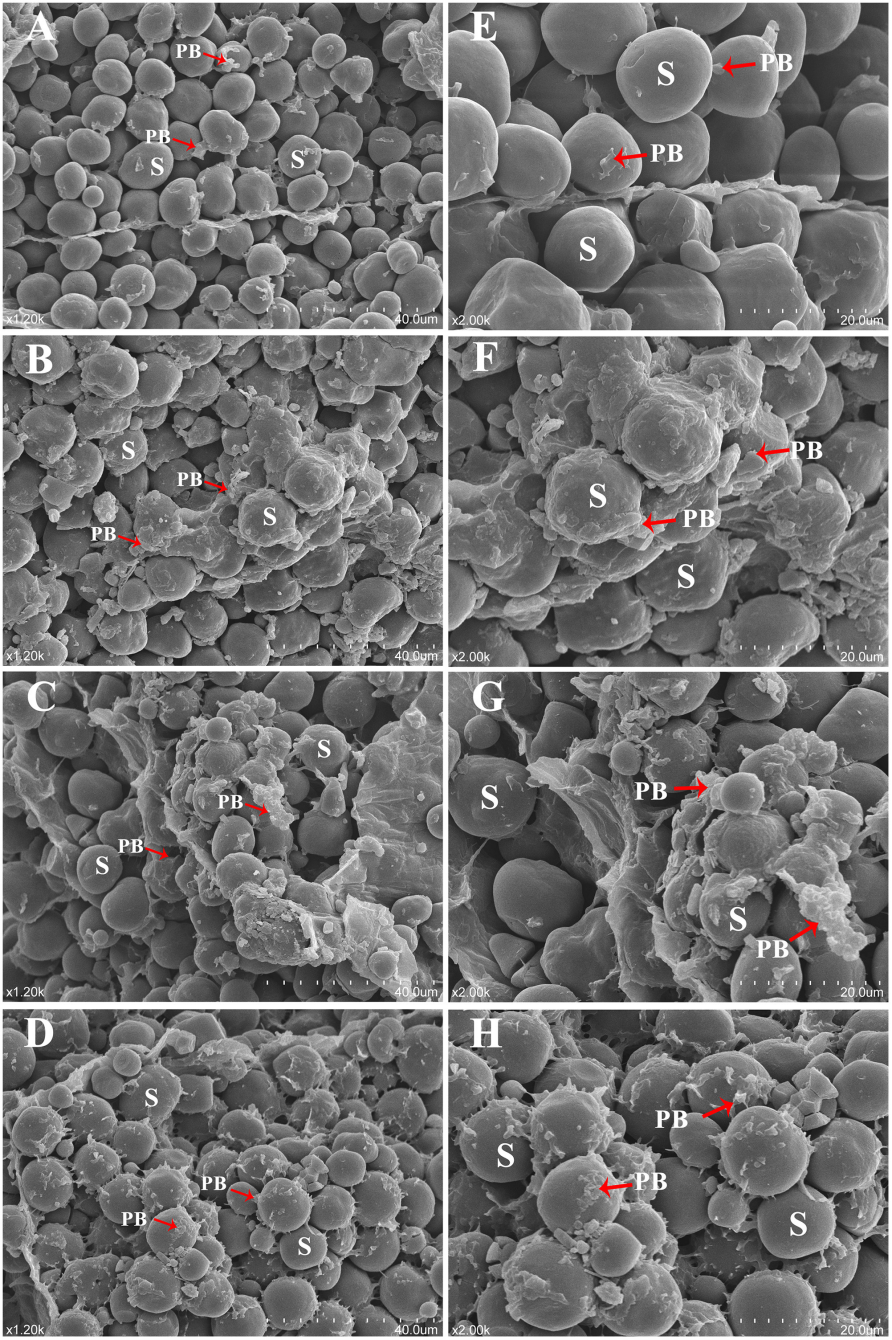
Scanning electron microscopy analysis of the central region of mature endosperm. (A, E) WT mature endosperm. (B, F) FA7 mature endosperm. (C, G) FA9 mature endosperm. (D, H) FA12 mature endosperm. Bar = 40 μm in (A)–(D), Bar = 20 μm in (E)–(H). PB: protein body, S: starch granule.

Mature transgenic T_6_ and WT endosperms were also analyzed by SEM. In the WT, cells in the central region of the starchy endosperm contained smooth starch grains with little proteinaceous matrix (Fig 5A and 5E). However, there was more proteinaceous matrix around the polygonal starch granules in the mature starchy endosperms of FA7, FA9 and FA12 (Fig 5B–5D and 5F–5H). These proteinaceous matrices, which mainly consisted of PBs, were increased in the mature transgenic endosperms compared with the WT.

These results suggested that *AtMAP18* improved the protein content by increasing PB numbers.

### Agronomic quality analysis

**Fig 6:**
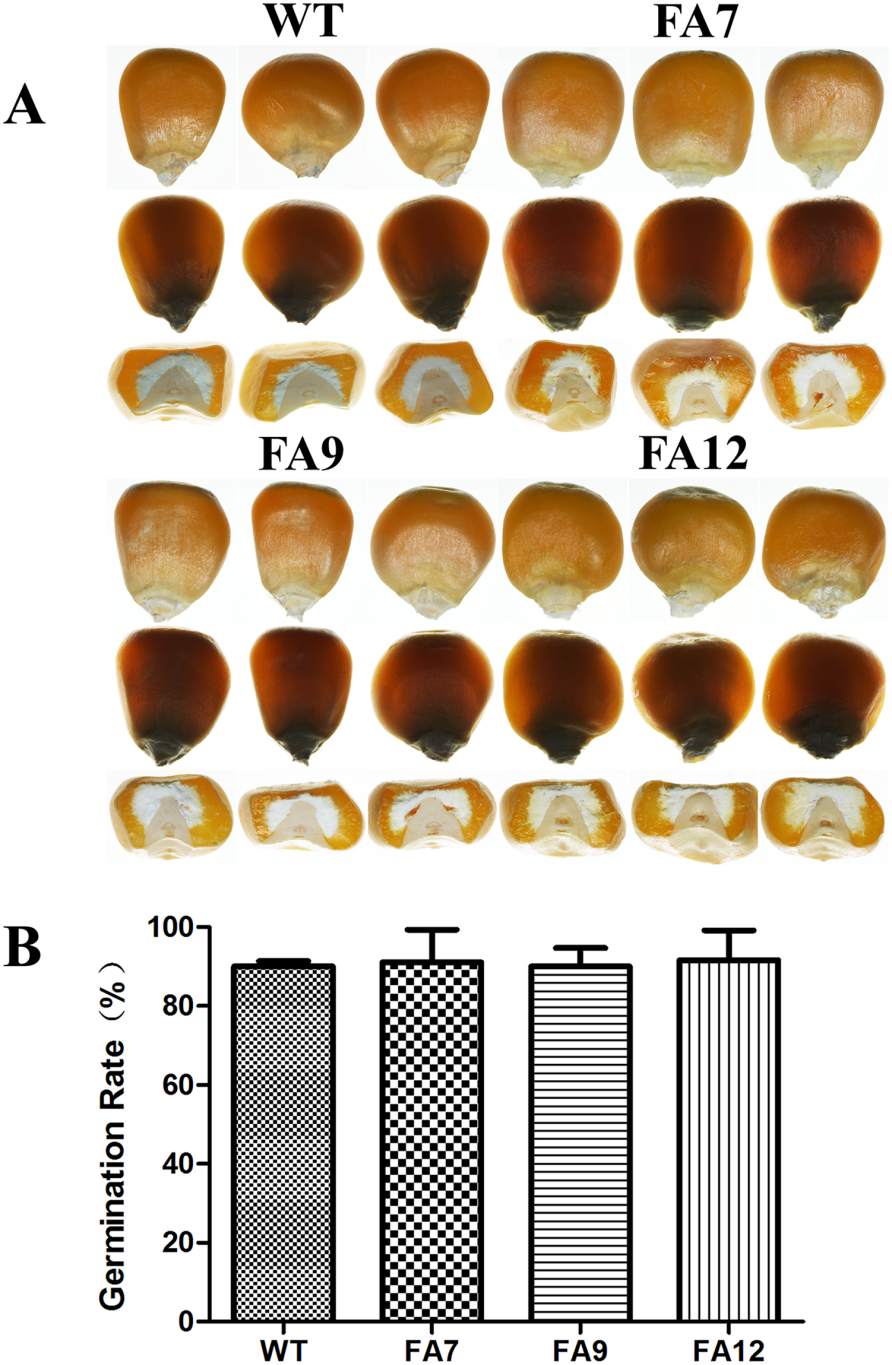
Kernel phenotype of T_6_ seeds in WT, FA7, FA9 and FA12. (A) Photographs of kernels were taken with incandescent light (Top and Bottom) and transmitted light (Middle). (B) Germination rate of T6 seeds. Student’s t-test was used to evaluate differences between each transgenic line and WT.

Kernel qualities and agronomic traits are very important in QPM breeding; thus, selected morphological features were analyzed in FA7, FA9, FA12 and the WT. Ear characters of the T_5_ and T_6_ homozygous transgenic generations were measured and no significant difference was found between the transgenic lines and WT (Tables 4 and 5). Kernel vitrification is a critical agronomic trait in maize. The vitreous phenotype of T_6_ transgenic maize kernels was observed using incandescent and transmitted light; kernel appearance was similar to the WT (Fig 6A). These results indicated that the increased protein and lysine contents in the transgenic lines did not affect the agronomic characters or kernel qualities. Additionally, the germination rate of transgenic seeds was also similar to WT (Fig 6B), implying the increased protein accumulation did not affected the seed development and maturation.

**Table 4:** Agronomic traits of T_5_ kernels in three transgenic lines.

Line	EL(cm)	BTL(cm)	ED(cm)	KW(g)	ER
FA7	11.24±1.45	0.97±0.54	36.15±0.92	24.23±1.81	12, 14
FA9	12.84±0.71	1.11±0.65	42.07±2.49	26.56±1.59	12, 14
FA12	10.33±1.52	0.68±0.38	31.68±2.49	23.83±2.67	12, 14
WT ^a^	11.29±1.21	1.34±0.63	33.38±2.77	23.72±5.24	12, 14

Values are means ± SD from the same lines in different generations. EL, ear length; BTL, bald tip length; ED, ear diameter; KW, 100-kernel weight; ER, numbers of ear rows.

^a^ WT: Hybrid 08×178 in the T_5_ generation as a control.

**Table 5:** Agronomic traits of T_6_ kernels in three transgenic lines.

Line	EL(cm)	BTL(cm)	ED(cm)	KW(g)	ER
FA7	11.00±1.36	0.73±0.16	39.49±1.39	27.43±2.16	12, 14
FA9	12.93±0.93	0.40±0.23	33.38±1.45	26.50±2.46	12, 14
FA12	10.43±0.67	0.66±0.57	35.70±2.23	26.92±0.82	12, 14
WT ^a^	11.48±2.27	0.60±0.33	34.62±0.67	25.09±1.62	12

Values are means ± SD from the same lines in different generations.

EL, ear length; BTL, bald tip length; EED, empty ear diameter; ER, numbers of ear rows; KW, 100-kernel weight; GER, grain number of ear row; ED, ear diameter

^a^ WT: Hybrid 08×178 in the T_6_ generation as a control.

To explore the potential use of *AtMAP18* transgenic maize, we obtained F_1_ hybrid lines by cross-breeding T_6_ transgenic maize with different wild type inbred lines. F_1_ maize inherited high lysine and protein contents from the transgenic maize. The protein content was increased up to 24.45% and the lysine content of three lines was also increased by more than 20% (Table 6). Additionally, the F_1_ hybrid lines showed good agronomic traits appropriate for production and application (Table 7). These results suggested that *AtMAP18* had the potential for commercial application in QPM breeding.

**Table 6:** Lysine and total protein contents of F_1_ kernels.

Line	Protein content (g/100 g seed)	Protein increase rate (%)	Lysine content (g/100 g seed)	Lysine increase rate (%)
FA7/FS	11.03±0.55*	8.16%	0.35±0.02***	12.49%
FA7/HBA58	11.06±0.77	8.46%	0.38±0.05**	22.82%
FA9/FS	11.21±0.77	9.93%	0.33±0.01**	7.26%
FA9/HBA58	12.69±0.34***	24.45%	0.34±0.04	10.04%
FA9/G33	11.18±0.48**	9.61%	0.39±0.02*	26.33%
FA9/RX87	11.17±0.74	9.54%	0.34±0.00	10.46%
FA9/HBA40	11.48±0.72*	12.53%	0.35±0.00***	14.18%
FA12/FS	11.30±0.51**	10.75%	0.37±0.01***	20.90%
FA12/HBA58	11.86±0.48***	16.25%	0.32±0.02	4.38%
WT ^a^	10.20±0.05	/	0.31±0.00	/

Values are means ± SD from the same lines in different generations.

^a^ WT: Hybrid 08×178 as a control.

* Significant difference between the transgenic lines and WT by Student’s *t*-test (* *p* < 0.05; ** *p* < 0.01; *** *p* < 0.001).

**Table 7:** Agronomic traits of F_1_ kernels in three transgenic lines.

Line	EL(cm)	BTL(cm)	ED(cm)	KW(g)	ER
FA7/FS	19.83±1.15	1.00±0.44	49.19±1.76	31.70±3.07	16,18
FA7/HBA58	21.13±0.83	0.55±0.54	52.33±0.63	37.68±2.91	16
FA9/FS	19.64±1.29	1.48±0.25	46.93±1.40	25.72±1.94	18,20
FA9/G86	18.92±0.14	1.02±0.14	53.14±1.66	35.40±2.26	16,18
FA9/HBA58	18.93±1.01	2.38±0.55	45.10±1.53	35.20±0.59	14
FA9/G33	18.67±1.01	2.38±0.55	49.14±1.53	28.65±0.59	18,20
FA9/RX87	17.48±2.40	1.95±0.52	47.60±1.24	32.21±2.52	16,18
FA9/HBA40	18.68±1.39	1.75±0.52	53.43±1.04	33.26±0.86	18,20
FA12/FS	19.62±2.12	2.50±0.49	46.55±2.01	28.13±1.90	18,20
FA12/HBA58	19.00±0.60	0.68±0.45	52.46±2.44	32.67±2.16	16,18,20

Values are means ± SD from the same lines in different generations.

EL, ear length; BTL, bald tip length; EED, empty ear diameter; ER, numbers of ear rows; KW, 100-kernel weight; GER, grain number of ear row; ED, ear diameter.

## Discussion and Conclusions

Based on the discovery of the high-lysine maize mutant *o2*, various approaches have been used to produce commercial high-yield, high-lysine maize. Genetic engineering is an effective way of obtaining high-lysine maize and should reduce the time needed to obtain commercial lines. Selectable marker genes are essential for plant genetic engineering and provide a powerful tool to determine the success of identify transformation events. However, several marker genes can impair the stability of the genetically engineered trait and induce unforeseen biosafety effects when left in the plant [29]. Therefore, the efficient production of ‘clean’ marker-free transgenic plants is necessary. There are several strategies to exclude selection genes to obtain marker-free plants in transgenic generations, such as co-transformation, site-specific recombination, multi-auto-transformation vectors, transposition systems and homologous recombination [29]. A binary vector with two T-DNAs containing the target gene and selection gene can also be used to get marker-free plants [22, 30]. In our study, a double T-DNA expression vector was used for the transformation and selection of transgenic plants. The *AtMAP18* and *hpt* genes were successfully separated in T1 plants and transgenic plants containing only the target gene (without the marker gene) were selected to generate progeny. This confirms that the double T-DNA vector system is an efficient way to get marker-free transgenic plants, and that it could be used in future research.

In previous research, seed-specific expression of the lysine-rich protein genes *Sb401* and *SBgLR* increased both the lysine and protein contents of maize seeds [19, 20]. AtMAP18 is a microtubule-associated protein with high-lysine content that contains the same K-K-E-E repeats as Sb401 and SBgLR. We expected that *AtMAP18* could also increase the protein and lysine contents of transgenic maize like *Sb401* and *SBgLR*. The results presented in this study show that the protein and lysine contents were obviously increased in transgenic lines containing *AtMAP18*. In T_1_ transgenic maize, the protein content was increased by 33.6% and the lysine content was also increased by 32.3% (Table 2). The protein and lysine contents of six T_1_ lines were improved by more than 20%, all of which were derived from parents with high lysine and protein contents. The lines with high protein and lysine contents (>20%) were selected to sow the next generation. So far, we have obtained three lines of T_6_ transgenic maize (FA7, FA9 and FA12). The protein contents were both increased by more than 20% in the (T_5_ and T_6_); kernels (Tables 2 and 3). Kernel qualities and agronomic traits are very important in QPM breeding. We also estimated the agronomic and quality traits of homozygous transgenic maize (T_5_ and T_6_); no significant differences were observed between the transgenic maize and WT (Tables 4 and 5, Fig 6). This evidence suggested that *AtMAP18* had a positive effect on the protein and lysine contents and that these characters were heritable in subsequent generations. Additionally, the increase of protein and lysine content in the transgenic lines had no effect on the agronomic characters or kernel qualities. This implied that *AtMAP18* transgenic maize had potential application in QPM breeding. Furthermore, we obtained F_1_ progeny by cross-breeding transgenic maize with different wild type inbred lines. Most of them inherited the high-lysine and high-protein characters and obtained features for commercial application, implying potential for future use.

We tried to explore the reason why *AtMAP18* increased the protein content of transgenic maize. AtMAP18 is a cytoskeleton-associated protein that has a destabilizing effect on cortical microtubules and influences actin organization. It binds to microtubules as well as microfilaments in *A*. *thaliana* [23, 24]. Our research indicated that *AtMAP18* increased both zein and non-zein proteins in the transgenic endosperm (Fig 2). Through ultrastructure observation, we also found that there were more PBs in the endosperm of transgenic maize than in the WT (Figs 4 and 5).

The cytoskeleton provides a spatial concentration of the translation machinery for efficient protein synthesis [31]. Many studies have proved that the cytoskeleton and associated proteins play an important role in protein synthesis and PB formation. For example, the accumulation of elongation factor 1A (eEF1A) contributed to a more extensive cytoskeletal network surrounding the RER, promoted synthesis of cytoskeleton-associated proteins, and increased the lysine content of the endosperm [32, 33]. eEF1A might also function in microtubule dynamics [31]. The cytoskeleton protein GAPDH, as a high-lysine containing protein, contributes significantly to the elevated level of lysine in the *o2* mutant, and might be associated with the expression of eEF1A [34]. There is mounting evidence that the cytoskeleton plays a crucial role in protein body biogenesis in plants [35].

Given all this, we hypothesize that AtMAP18 promotes microtubule activity and microfilament organization in the transgenic endosperm. Subsequently, it plays a role in the synthesis and organization of zeins, leading to PB assembly. At the early stage of endosperm development, microtubules and microfilaments are organized into a dense network before starch and storage protein deposition [36]. The cytoskeleton, in particular the microfilament system, serves as an attachment site for polysomes [37]. Zeins are synthesized by these polysomes bound to the RER [36]. Thus, AtMAP18 may connect polysomes more efficiently and increase translational activity. When PBs and starch granules begin to accumulate, more PBs are formed in the transgenic endosperm than in the WT, and the growth of PBs in the endosperm limits the development of starch granules at this stage. This is a reasonable explanation for the reduced starch granules in the transgenic endosperm (Fig 4). PBs comprise a large group of zeins and are enmeshed in an extensive cytoskeleton network. Here, the starch granules were surrounded by more PBs bound to the cytoskeleton (Fig 5), which led to increased zein content in the transgenic endosperm (Fig 3). Increased expression of the high-lysine protein AtMAP18 resulted in elevated levels of lysine in the transgenic endosperm. However, it is impossible that the lysine content increased by more than 20% merely through the accumulation of AtMAP18. Cytoskeleton proteins are major contributors to the lysine content of maize [35]. AtMAP18 may increase the accumulation of cytoskeleton proteins, hence, improving the lysine content in transgenic seeds. Moreover, a previous study showed that cytoskeleton-associated proteins increased with the production of cytoskeleton proteins during development, and most of these proteins were relatively high in lysine [35]. Thus, we conclude that AtMAP18 may contribute to an increase of cytoskeleton-associated proteins. Consequently, the non-zein and total protein contents are increased as well (Fig 3). This is a possible explanation for the function of AtMAP18. However, further investigation is necessary to understand the mechanism by which AtMAP18 improves the lysine and protein contents in transgenic maize seeds.

AtMAP18, Sb401 and SBgLR are all microtubule-associated proteins with high-lysine content, but they have different roles in regulating microtubule organization. Sb401 and SBgLR are homologous proteins from potato that bind to MTs and enhance tubulin polymerization [28, 38], while AtMAP18 has an inhibitory effect on tubulin polymerization, and severs and disassembles microtubules *in vivo* [24]. This study and previous work indicates that the expression of AtMAP18, Sb401 or SBgLR is correlated with increased levels of lysine and total protein in maize seeds. However, the functional mechanism may be different between AtMAP18 and SB401. Therefore, future research should be aimed at investigating this.

In conclusion, our results reveal that the lysine-rich gene *AtMAP18* plays an obvious role in enhancing the nutritional quality of maize. This also suggests a potential commercial application for *AtMAP18* in cultivating valuable maize with high protein, high lysine and hard endosperm traits. We made a preliminary exploration into the reasons why *AtMAP18* increased the protein content of transgenic maize, and put forward a hypothesis for how *AtMAP18* works in maize endosperm cells. This article provides evidence to support the view that the cytoskeleton and associated proteins play an important role in protein synthesis and PB formation.

## Supporting Information

S1 Table Copy number assay of T_2_ transgenic lines by relative quantitative PCR. Values are means ± SD from three experiments. ^a^ A maize gene *glutamic acid and Lysine rich* (*ZmGLR*, GRMZM2G123558) was selected as a reference gene. *ZmGLR* was a single-copy endogenous gene from maize.
(DOCX)
