# Supplementary material for: Seed-Specific Expression of the Arabidopsis AtMAP18 Gene Increases both Lysine and Total Protein Content in Maize
Source: PLoS One. 2015 Nov 18;10(11):e0142952. doi: 10.1371/journal.pone.0142952 (PMC4651559; doi:10.1371/journal.pone.0142952)
Supplement: S1 Table — Values are means ± SD from three experiments. a A maize gene glutamic acid and Lysine rich (ZmGLR, GRMZM2G123558) was selected as a reference gene. ZmGLR was a single-copy endogenous gene from maize. (DOCX) [file pone.0142952.s001.docx]

| line | *At168 Ct* | ^a^*ZmGLR Ct*  (reference) | Relative copy number  (AtMAP18/ZmGLR) | Copy number |
| --- | --- | --- | --- | --- |
| FA3 | 21.71±0.19 | 22.60±0.11 | 1.86±0.20 | 2 |
| FA7 | 23.44±0.16 | 23.75±0.14 | 1.24±0.14 | 1 |
| FA9 | 23.40±0.14 | 23.40±0.13 | 1.00±0.10 | 1 |
| FA12 | 22.41±0.02 | 22.35±0.20 | 0.96±0.01 | 1 |
| FA14 | 21.54±0.08 | 22.56±0.14 | 2.02±0.11 | 2 |
